# Comparing the Efficacy of Transforaminal and Caudal Epidural Injections of Calcitonin in Treating Degenerative Spinal Canal Stenosis: A Double-Blind Randomized Clinical Trial

**DOI:** 10.5812/aapm-142822

**Published:** 2024-02-16

**Authors:** Poupak Rahimzadeh, Farnad Imani, Reza Farahmand Rad, Seyed Hamid Reza Faiz

**Affiliations:** 1 Department of Anesthesiology and Pain Medicine, Pain Research Center, School of Medicine, Iran University of Medical Sciences, Tehran, Iran; 2 Department of Anesthesiology and Pain Medicine, Minimally Invasive Surgery Research Center, School of Medicine, Iran University of Medical Sciences, Tehran, Iran

**Keywords:** Calcitonin, Spinal Canal Stenosis, Clinical Trial, Back Pain, Caudal Epidural, Transforaminal

## Abstract

**Background:**

Lumbar spinal stenosis (LSS) is the most common indication for lumbar surgery in elderly patients. Epidural injections of calcitonin are effective in managing LSS.

**Objectives:**

This study aimed to compare the efficacy of transforaminal and caudal injections of calcitonin in patients with LSS.

**Methods:**

In this double-blind randomized clinical trial, LSS patients were divided into two equal groups (N = 20). The first group received 50 IU (international units) of calcitonin via caudal epidural injection (CEI), and the second group received 50 IU of calcitonin via transforaminal epidural injection (TEI). The Visual Analogue Scale (VAS) and Oswestry Low Back Pain Disability Questionnaire (ODI) were used to assess the patient's pain and ability to stand, respectively. Visual Analogue Scale and ODI scores were recorded and analyzed.

**Results:**

The results showed that caudal and TEIs of calcitonin significantly improved pain and ability to stand during follow-up compared to before intervention (P < 0.05). Additionally, CEI of calcitonin after 6 months significantly reduced pain in LSS patients compared to TEI of calcitonin (P < 0.05). However, no significant difference was observed between the two epidural injection techniques in improving the patient's ability to stand (P > 0.05).

**Conclusions:**

The results of the study indicate that epidural injection of calcitonin in long-term follow-up (6 months) had a significant effect on improving pain intensity and mobility in patients with LSS, and its effect on pain in the TEI method was significantly greater than that in the CEI method.

## 1. Background

Lumbar spinal stenosis (LSS) stands as a primary cause of back pain and disability, marked by the narrowing of the spinal canal leading to the compression of neural structures by surrounding soft tissues and bones (1). While LSS can be congenital, it is primarily instigated by degenerative factors such as spondylolisthesis and age-related changes, encompassing disc degeneration, hypertrophy of the ligamentum flavum, and complications related to facet joints (2). Notably, LSS ranks among the most common reasons for lumbar surgery in elderly patients (3, 4), affecting roughly 27.2% of the population (5, 6).

In 1994, it was estimated that the United States annually spent $1 billion on LSS surgery (7). Despite being frequently recommended, surgery poses potential complications, particularly for elderly patients (3, 8). Consequently, conservative management options such as physiotherapy, medication, and non-surgical interventions like epidural injections are often initially considered (9-13). The goal of epidural injections is to alleviate pain and enhance function through various mechanisms, including anti-inflammatory effects (12-14).

Numerous studies have demonstrated the effectiveness and cost-effectiveness of epidural injections in managing LSS pain (15, 16). Local anesthetics and corticosteroids are commonly used in these injections (17), and calcitonin, a hormone, is another medication used specifically for LSS epidural injections (18). Calcitonin is primarily used to treat conditions like Paget's disease, hypercalcemia due to cancer, acute bone loss owing to sudden immobility, and postmenopausal osteoporosis. Calcitonin has been effective in enhancing myelin regeneration by reducing ischemia and venous congestion. It stops osteoclastic activity and encourages ossification by hindering calcium uptake from bones and promoting osteoblastic action. It has shown beneficial effects in controlling pain and improving function and movement in LSS patients (18, 19).

A review article by Manchikanti et al. in 2014 highlighted that different epidural injection techniques (interlaminar, transforaminal, and caudal) have varying effectiveness in treating LSS. The caudal and interlaminar methods have shown long-term effectiveness and are more efficient compared to the transforaminal method (17). Therefore, the choice of epidural injection technique plays a crucial role in the treatment of LSS.

## 2. Objectives

This study aimed to compare the therapeutic efficacy of transforaminal and caudal epidural injections (CEIs) of calcitonin in LSS patients. The primary outcome was pain scores in both groups.

## 3. Methods

### 3.1. Study Design and Patients

**Figure 1.**
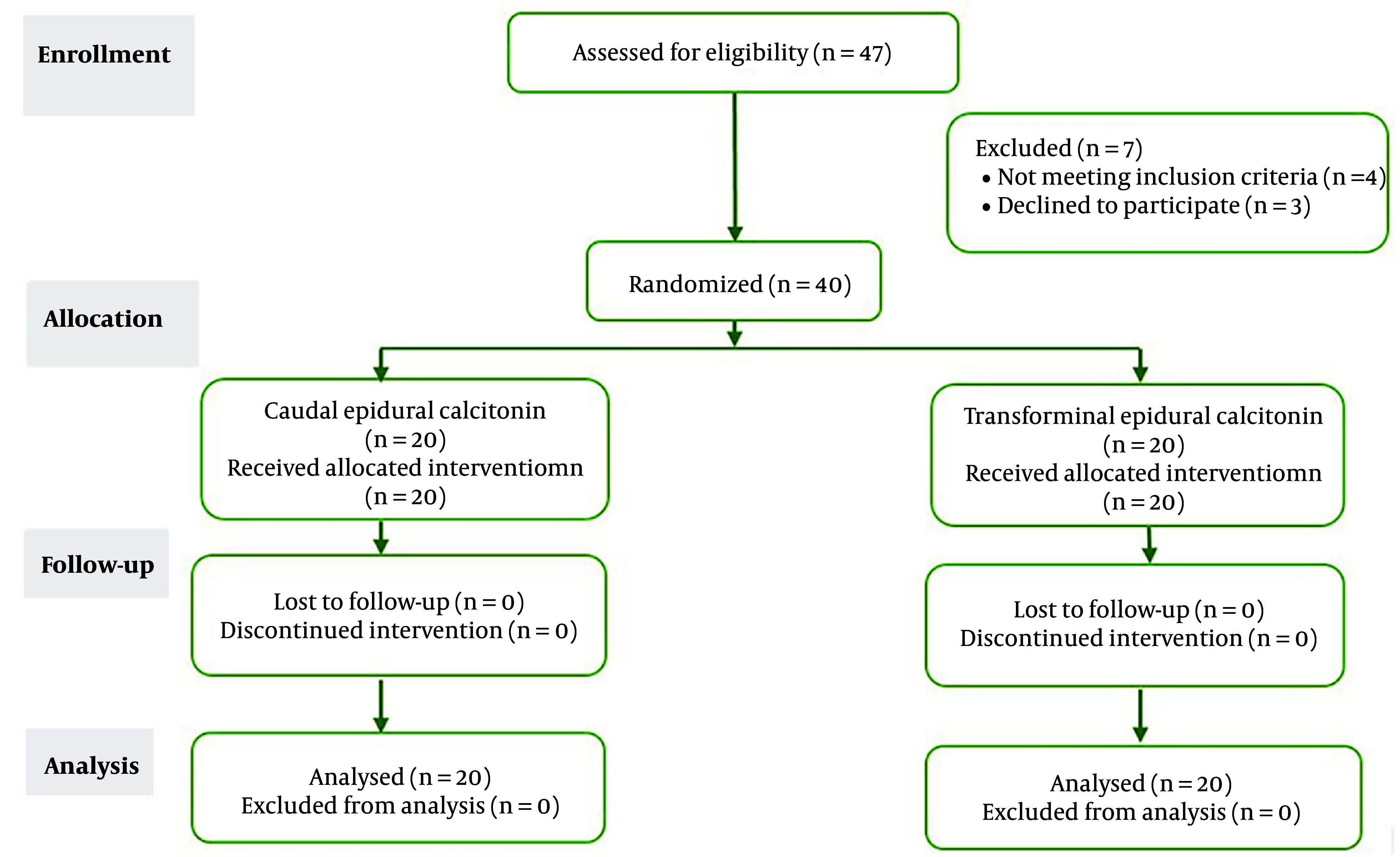
The CONSORT flow chart presents clinical trials.

This study was a double-blind, randomized clinical trial conducted at the pain clinic of Hazrat Rasool Akram Hospital. A total of 40 LSS patients who had received calcitonin injections into the caudal epidural and transforaminal epidural space were included. The patients were divided into two groups, with each group consisting of 20 participants. The study was designed and conducted in accordance with the consolidated standards of reporting trials (CONSORT) guideline (Figure 1). 

The research followed the principles of the Declaration of Helsinki, and it was approved by the Ethics Committee of the Iran University of Medical Sciences (ethics code: IR.IUMS.REC.1400.092). Written informed consent was obtained from all participants before any intervention. The study was assigned a clinical trial code (IRCT20120814010599N29).

The study utilized block randomization to assign patients into two groups (group A and group B). A senior nurse placed the codes of the first 20 patients in one envelope and the codes of the next 20 patients in another envelope. The nurse was not involved in patient follow-up. The senior resident who conducted assessments and collected data was unaware of the group assignments. The pain specialist who administered the interventions was also unaware of the groupings and materials used.

### 3.2. Eligibility Criteria

- Patients over 40 years old with a history of chronic low back pain, with or without lower extremity pain, rated as more than 4 based on the Visual Analogue Scale (VAS) pain measurement criteria.

- Patients with a minimum of 3 months of pain experience and a diagnosis of central spinal stenosis due to positive neurogenic intermittent claudication, confirmed by MRI findings (anterior-posterior diameter canal < 12 mm at the level of the lumbar vertebrae), with or without radicular pain.

### 3.3. Exclusion Criteria

- International normalized ratio (INR) > 1.5

- Platelet count < 50 000

- Needle site infection

- Congenital spinal canal stenosis

- Degenerative spondylolisthesis

- Psychiatric disorders affecting cooperation

- History of spinal surgery

- Previous chronic opioid use

- Peripheral vascular disease

- Uncontrolled disease (diabetes or hypertension)

- History of adverse reactions to anesthetics, steroids, or calcitonin.

### 3.4. Patient Grouping and Intervention

The study randomly assigned 40 eligible patients into two groups of 20:

- Caudal epidural injection group: Patients in this group received a 50 IU calcitonin injection via CEI.

- Transforaminal epidural injection (TEI) group: Patients in this group received a 50 IU calcitonin injection via TEI.

### 3.5. Preparing Patients for Epidural Injection of Calcitonin

Before the procedure, a 20 G intravenous cannula was inserted into the patient's vein for medication delivery, including crystalloid and sedation. Midazolam (0.05 mg/kg) was administered to the patient. Initial non-invasive monitoring of arterial blood pressure, electrocardiogram, and pulse oximetry was performed for each patient.

### 3.6. Transforaminal Epidural Injection of Calcitonin

The procedure was performed under sterile conditions while the patient was in the prone position. The transforaminal injection was performed based on the entry of the spinal nerve root into the foramen at the level of spinal canal stenosis. A 22-gauge needle (Bella-D Coudé^®^) was inserted into the stenosis of the spinal canal, followed by an injection of 0.5 mL of contrast media (omnipaque 300 mg/mL) and, if necessary, additional contrast media before the injection of ropivacaine 0.2% 4 mL plus 25 IU calcitonin in the transforaminal space on each side. A fluoroscope was used to increase the accuracy and optimality of exposure.

### 3.7. Caudal Epidural Injection of Calcitonin

The procedure was conducted in a sterile environment, with the patient lying prone. The sacral hiatus was identified, and 3 mL of lidocaine was applied to the skin and ligament covering it. Vital signs were continuously monitored. A 19-gauge Tuohy needle was inserted at a 45-degree angle between the sacral horns into the sacral hiatus, following an upward path until it reached the epidural space through the sacrococcygeal ligament. An aspiration test and a “hoosh” test were performed to confirm proper needle placement (20), and placement was verified using a C-arm with lateral and anterior-posterior views after injecting contrast. Subsequently, ropivacaine 0.2% (8 mL) plus 50 IU of calcitonin was injected.

### 3.8. Patient Follow-up and Data Collection

Demographic and baseline information of patients were recorded before the intervention. After cleaning the injection site and dressing, all patients were transferred to the recovery room and monitored for 2 hours. They were discharged in case of stable condition and vital signs. Pain experienced during movement was measured and recorded at five-time points: Before the intervention, at the second week, and at the first, third, and sixth months after the intervention. The patient's ability to stand was measured and recorded at four-time points: Before the intervention, in the second week, and in the first and third months after the intervention. Pain assessment during movement was documented using the VAS, ranging from 0 to 10 (0 means no pain, 10 means worst possible pain). The patient's ability to stand was assessed using the standard Oswestry Low Back Pain Disability Questionnaire (ODI), which comprised 10 sections, with each section containing 6 sentences scored from 0 to 5 (21, 22).

### 3.9. Statistical Analysis

Data from all patients in the two groups were analyzed using SPSS software version 22. Central indicators (mean and standard deviation) were used for quantitative variables, while frequency (%) was used for qualitative variables. A significance level of P < 0.05 was considered statistically significant.

## 4. Results

Initially, 47 patients were included in the study, but 7 patients were later excluded (4 due to lack of inclusion criteria and 3 due to dissatisfaction with participation). Finally, 40 patients with LSS were randomly divided into two groups (N = 20), receiving either Caudal epidural or TEI of calcitonin. No participants were excluded during the intervention and follow-up, and data from all patients were included in the statistical analysis (Figure 1). 

### 4.1. Demographic Results

Twenty participants were included in each group. The mean age of patients was 59.7 ± 13.7 years. Out of the 40 participants, 17.5% were male and 82.5% were female. In the TEI group, 15 patients (75%) were female, while in the CEI group, 18 patients (90%) were female. There was no significant difference in sex distribution between the two groups (P = 0.04). The mean weight of patients in the TEI and CEI groups was 67.65 ± 7.95 kg and 69.2 ± 7.85 kg, respectively, with no statistically significant difference observed between the two groups (P = 0.28). Similarly, there were no significant differences between the two groups in terms of baseline variables (P > 0.05).

### 4.2. Comparison of Pain Scores and ODI Scores

In the TEI group, a significant decrease in mean VAS and ODI scores was observed at different follow-up stages (P < 0.05). Two weeks after TEI of calcitonin, there was a statistically significant decrease in mean VAS and ODI scores (P < 0.05) (Table 1). Similarly, in the CEI group, a significant decrease in mean VAS and ODI scores was observed at different follow-up stages (P < 0.05). Two weeks after the caudal epidural injection of calcitonin, there was a statistically significant decrease in mean VAS and ODI scores (P < 0.05) (Table 2). 

**Table 1.** Comparison of Changes in the Mean Pain Score and ODI of Patients in Different Stages of Follow-up in Transforaminal Epidural Injection

Variables	Transforaminal	P-Value
**VAS score **		0.001
Before	4.5 ± 0.51	
Week 2	2.01 ± 0.97	
Month 1	1.06 ± 1.04	
Month 3	0.7 ± 0.66	
Month 6	0.31 ± 0.47	
**ODI score **		0.001
Before	31.15 ± 7.49	
Week 2	12.10 ± 6.4	
Month 1	4.45 ± 3.87	
Month 3	2.23 ± 1.85	

Abbreviations: VAS, visual analogue scale; ODI, Oswestry Disability Index.

**Table 2.** Comparison of Changes in Mean Pain Score and Oswestry Disability Index of Patients in Different Stages of Follow-up in Caudal Epidural Injection

Variables	Caudal	P-Value
**VAS score **		0.001
Before	4.25 ± 0.39	
Week 2	2 ± 2.05	
Month 1	1.54 ± 0.55	
Month 3	0.8 ± 0.52	
Month 6	0.04 ± 0.22	
**ODI score **		0.001
Before	32.15 ± 8.06	
Week 2	13.35 ± 4.15	
Month 1	5.25 ± 3.07	
Month 3	1.19 ± 0.8	

Abbreviations: VAS, visual Analogue scale; ODI, Oswestry Disability Index.

### 4.3. Comparison of Changes in Mean Pain Score

Initially, there was no significant difference in mean VAS scores between the two groups before the intervention (P = 0.28). However, at the 6-month follow-up, the mean pain score in the CEI group (0.04 ± 0.22) was significantly lower than that in the TEI group (0.31 ± 0.47) (P = 0.041). No significant differences in mean pain scores were observed between the two groups at other follow-up stages. In summary, only at the sixth month after treatment did patients receiving a caudal epidural injection of calcitonin have a lower mean pain score. Detailed results comparing pain scores at different treatment stages in the two groups can be found in Table 3. 

**Table 3.** Comparison of Trend of Changes in Mean Pain Score of Patients in Different Stages of Treatment in the Two Groups

Variables	Transforaminal	Caudal	P-Value
**VAS score **			
Before	4.5 ± 0.51	4.25 ± 0.39	0.28
Week 2	2.01 ± 0.97	2 ± 2.05	0.98
Month 1	1.06 ± 1.04	1.54 ± 0.55	0.95
Month 3	0.7 ± 0.66	0.8 ± 0.52	0.59
Month 6	0.31 ± 0.47	0.04 ± 0.22	0.041

Abbreviation: VAS, visual Analogue scale.

The mean ODI score before the intervention did not show a significant difference between the two groups (P = 0.25). Similarly, there were no statistically significant differences in the mean ODI scores at any follow-up stage between the two groups (P > 0.05). Detailed results comparing the ODI scores at different follow-up stages in the two groups can be found in Table 4. 

**Table 4.** Comparison of Trend of Changes in Mean Oswestry Disability Index Score of Patients in Different Stages of Treatment in the Two Groups

Variables	Caudal	Transforaminal	P-Value
**ODI score **			
Before	31.15 ± 7.49	32.15 ± 8.06	0.68
Weeks 2	12.10 ± 6.4	13.35 ± 4.15	0.46
Month 1	4.45 ± 3.87	5.25 ± 3.07	0.47
Month 3	2.23 ± 1.85	1.19 ± 0.8	0.074

Abbreviation: ODI, Oswestry Disability Index.

## 5. Discussion

In this study, our aim was to compare the effectiveness of administering calcitonin via transforaminal and caudal routes of injection in providing pain relief and reducing movement disabilities in patients with LSS. Both transforaminal and caudal epidural injections of calcitonin demonstrated efficacy in reducing pain and inability to stand. However, after a six-month follow-up, caudal epidural injection was found to be more effective in reducing pain compared to transforaminal injection. Previous studies have explored the use of calcitonin for LSS treatment, including subcutaneous, intramuscular, and inhalation administration (23, 24). A study by Elsheikh and Amr in 2016 introduced the epidural injection of calcitonin for LSS patients, demonstrating significantly lower pain intensity that was sustained even after one year. This injection also improved the ODI score, indicating an improvement in the inability to stand (18).

The exact mechanism by which calcitonin manages pain and improves the ODI score in LSS patients remains unknown. However, it is believed that calcitonin acts as a pain-relieving agent, directly impacting bone and nerve tissue in the LSS area through the release of B-endorphin, which induces analgesic effects (25). Calcitonin has been shown to induce cAMP formation in chondrocytes in animal models, inhibit type II collagen degradation, and enhance metalloproteinase matrix activity, which may lead to a decrease in the osteoarthritis process (26). Additionally, calcitonin directly affects nerve tissue by eliminating venous congestion, reducing ischemia, and enhancing myelin regeneration (27).

Comparing injection techniques, caudal and interlaminar epidural injections were found to be more effective than the transforaminal method for long-term pain relief in patients (17). The caudal method, allowing for greater absorption of calcitonin in the epidural space, was particularly effective in reducing pain in patients with LSS. However, the transforaminal method may be more suitable for patients with radicular pain due to its direct access to the involved nerve and dorsal root ganglion (28). The volume of material used for injection was also noted to be greater in the caudal method compared to the transforaminal method (29).

### 5.1. Conclusions

In line with previous findings by Elsheikh and Amr (18), our study suggests that epidural injection of calcitonin has a significant effect on improving pain intensity and mobility in patients with LSS during long-term follow-up (6 months). Notably, the caudal technique was found to be remarkably more effective than the transforaminal technique in reducing pain. We suggest that epidural calcitonin may be considered a novel treatment method for pain management in spinal stenosis.

## Clinical Trial Registration Code:

IRCT20120814010599N29.
